# Bile acid triggered release of daunorubicin from silica nanoparticle-stabilized antibubbles

**DOI:** 10.1016/j.ijpx.2026.100632

**Published:** 2026-08-09

**Authors:** Rabia Zia, Annemarije van der Vorst, Albert T. Poortinga, Akmal Nazir, Cornelus F. van Nostrum

**Affiliations:** a Department of Pharmaceutics, Utrecht Institute for Pharmaceutical Sciences, Utrecht University, Utrecht, the Netherlands; b Department of Pharmacology and Therapeutics, College of Medicine and Health Sciences, United Arab Emirates University, Al Ain, United Arab Emirates; c Department of Mechanical Engineering, Polymer Technology, Eindhoven University of Technology, Eindhoven, the Netherlands; d Department of Food Science, College of Agriculture and Veterinary Medicine, United Arab Emirates University, Al Ain, United Arab Emirates

**Keywords:** Oral drug delivery, Daunorubicin, Antibubbles, Silica nanoparticle, Bile salt-triggered release

## Abstract

We report on the preparation and release behavior of daunorubicin-loaded antibubbles targeted for intestinal drug release. The antibubbles were prepared from a Pickering-stabilized W_1_/O/W_2_ double emulsion, then freeze-dried and rehydrated to create the distinct structure of antibubbles, i.e., water-in-air-in-water (W_1_/A/W_2_). Daunorubicin was loaded into the inner aqueous phase, surrounded by two particle-stabilized interfaces separated by an air gap. Fumed silica nanoparticles with varying degrees of hydrophobicity were used to stabilize both interfaces. The antibubbles, when rehydrated, had a mean diameter of approximately 25 μm and a drug entrapment efficiency of >90%. The release profile showed a non-significant release of the drug in both acidic and neutral media. Notably, a burst release of the drug (around 60%) was observed when the antibubbles were exposed to bile salts in simulated intestinal conditions. Hence, the bile salts were a major driving force in dislocating the nanoparticles at the interfaces. We have supported our findings with quantitative drug-release measurements (measured spectrophotometrically) and microscopic observations of structural changes across different media. Furthermore, protection of the encapsulated daunorubicin from degradation was investigated when the antibubbles were exposed to pH 9 or above (at which daunorubicin typically degrades). The antibubbles preserved almost all the drug over 24 h, whereas most of it (∼90%) degraded in the unencapsulated form. Based on these initial results, antibubbles seem promising for delivering drugs to the intestine, as a proof-of-concept for oral chemotherapy.

## Graphical abstract

**Figure:**
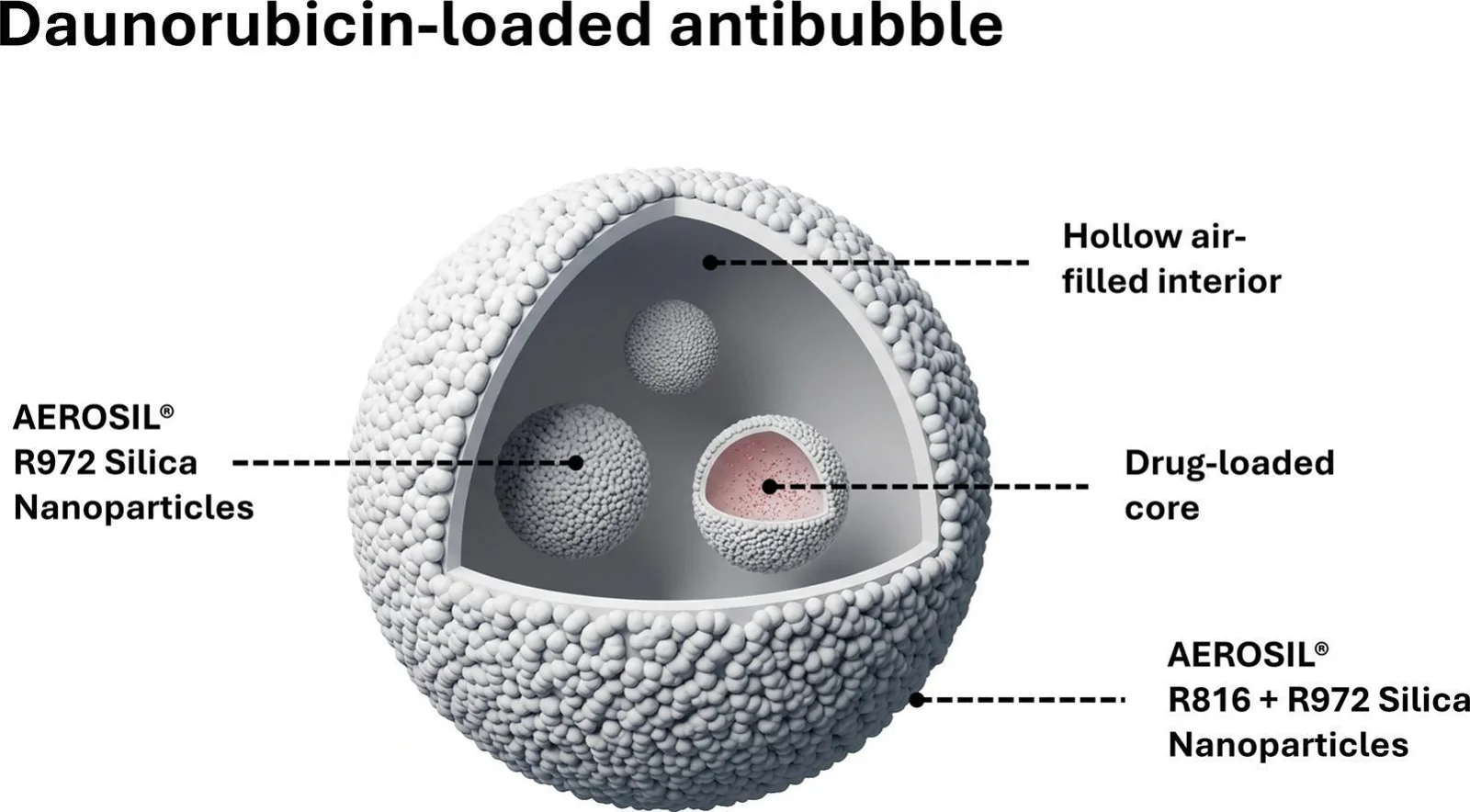

## 1 Introduction

Oral chemotherapy is an exemplary drug administration route with a high interest in pharmaceutical research and development. Oral drug administration has several benefits, such as i) patient compliance, ii) cost-effectiveness, iii) prevention of discomfort and pain linked with the use of needles in intravenous therapy, iv) reduction of additional hospitalization, etc. (Borner et al., 2001; Gornas and Szczylik, 2007; Liu et al., 2016). However, a drug must effectively pass through several barriers that can reduce oral bioavailability. Some of the most common issues resulting in low drug bioavailability are: i) poor drug solubility and permeability, ii) strong acidic conditions in the stomach, iii) digestive enzymes and natural flora of the gastrointestinal (GI) tract causing drug degradation, iv) mucus barriers preventing the drug absorption, and v) efflux transporters, such as P-glycoprotein (P-gp) and multidrug resistance proteins (MRPs), pumping drugs back into the intestinal lumen after they have entered intestinal cells (Huang et al., 2015; Mazzaferro et al., 2013; Pathak and Raghuvanshi, 2015). For instance, anticancer drugs such as paclitaxel and doxorubicin have an extremely low oral bioavailability of not more than 1% and 5%, respectively (Mei et al., 2013). Some rational attempts to increase bioavailability by encapsulating chemotherapeutics for oral drug delivery in a dispersion formulation include nano-emulsions, dendrimers, micelles, liposomes, solid-lipid nanoparticles, and cubosomes (Mei et al., 2013; Pimenta et al., 2023; Sohail et al., 2018). Furthermore, delivering the drug safely to a specific segment of the GI tract (e.g., the small intestine) for maximum drug absorption or site-specific delivery of chemotherapeutics remains a key interest for researchers.

Daunorubicin (a red pigment) is an anthracycline glycoside produced as a secondary metabolite by *Streptomyces peucetius* (Vasanthakumar et al., 2013). It is a chemotherapeutic agent used for the treatment of certain types of cancer, such as acute lymphoblastic leukemia (ALL), acute myeloid leukemia (AML), and chronic myelogenous leukemia (CML) (Grein, 1987; Thirumaran et al., 2007). The proposed mechanism of action is interference with DNA, topoisomerase I and II, entrapment of reactive oxygen species, and induction of apoptosis (Ahmad et al., 2019). Commercially, the drug is only available in the form of injections, e.g., DaunoXome® and VYXEOS™. However, intravenous therapy can never completely overcome severe side effects like cardiotoxicity, myelosuppression, tissue death at the site of injection, ulcers, and multi-drug resistance due to high doses (Ahmad et al., 2019). Nevertheless, oral chemotherapy is a less preferred option due to the drug's low bioavailability. Various oral formulations of daunorubicin have been tested in the past to cope with this. However, these formulations are still in the experimental phase, and to our knowledge, none have been commercially available until now. In the broader context of oral anthracycline delivery, most formulation studies have focused on doxorubicin rather than daunorubicin. These include polymeric or polysaccharide-based carriers, chitosan-coated nanoparticles or liposomes, lipid-based nanocarriers, and self-nanoemulsifying systems that have been designed to reduce premature gastric release, improve intestinal stability or permeability, and enhance oral bioavailability (Benival and Devarajan, 2012; Qin et al., 2024; Su et al., 2016; Usmani et al., 2019; Wu et al., 2020). However, direct studies on oral daunorubicin formulations remain comparatively limited (Ahmad et al., 2019; Lam et al., 2014a), which leaves scope for alternative delivery systems that can protect daunorubicin during gastric transit and release it in the intestinal environment.

To improve bioavailability by achieving the highest possible concentration of the free drug in the intestine (below the dose-limiting toxicity to the gastrointestinal epithelium), stable encapsulation is required during transit from the mouth to the stomach to prevent (acidic) degradation, together with a rapid burst release upon entering the intestine. Simultaneously, masking the drug's bad taste, and protecting the GI tract against high concentrations of chemotoxic drugs are also required. If ingested directly, the free drug can cause mouth ulcers and local tissue necrosis when contacting the mucus membranes directly (Ahmad et al., 2019; Lu et al., 2009; Wang et al., 2011).

In this study, we propose a novel encapsulation system, i.e., nanoparticle-stabilized antibubbles, for daunorubicin's oral/intestinal delivery. The antibubbles contain liquid core(s) surrounded by an air shell in a bulk liquid, i.e., in the form of a water-in-air-in-water dispersion (Zia et al., 2024). Such antibubbles are the freeze-dried and reconstituted product of particle-stabilized double emulsions (water-in-oil-in-water) produced using cryoprotecting sugars (e.g., maltodextrin) in the aqueous phases and a volatile oil (Poortinga, 2013; Zia et al., 2022). The double emulsion is frozen (preferably via quick freezing), followed by freeze-drying to remove the water and volatile oil. The freeze-dried powder, on rehydration of the sugar-containing compartments, will give the required antibubbles. The cryoprotectant prevents the collapse of the structure during freeze-drying, maintains the structure's integrity, and attracts water upon rehydration. The hydrophilic drug (such as daunorubicin) can be incorporated inside the liquid cores. Previously, we developed antibubbles that degrade and release their contents at low pH (e.g., within the stomach) (Zia et al., 2023). However, further research is needed to develop antibubbles that can release the drug in the intestine, e.g., which become unstable in response to external stimuli inside the GI tract. Therefore, the focus of this study was to produce daunorubicin-loaded antibubbles to protect the drug at low pH (i.e., stomach) and trigger its release in the intestinal environment, rather than to act as an absorbable particulate carrier.

## 2 Materials and methods

### 2.1 Materials

Daunorubicin hydrochloride (European Pharmacopoeia reference standard) was supplied by Fagron BV (The Netherlands). The hydrophobized fumed silica particles AEROSIL® R972 and AEROSIL® R816 were supplied by Evonik Gulf FZE (Dubai, UAE), with AEROSIL® R816 being the least hydrophobic, as indicated by its carbon content. Glucidex® 9 (potato maltodextrin, DE: 9) was manufactured by Roquette (France) and provided by Azelis (Belgium). Cyclohexane (≥99.5%), d-mannitol (≥98%), sodium chloride (≥99.5%), pepsin (from porcine gastric mucosa, 3200–4500 units/mg), pancreatin (from the porcine pancreas), sodium taurocholate hydrate (≥97%), sodium phosphate monobasic (≥99.5%), Bile extract (≥97%), and Tween® 20 (polysorbate 20) were supplied by Sigma-Aldrich (Germany). Certipur® buffer solutions: pH 2 (citric acid/sodium hydroxide/hydrogen chloride), pH 4 (citric acid/sodium hydroxide/hydrogen chloride), pH 7 (di‑sodium hydrogen phosphate/potassium dihydrogen phosphate), and pH 9 (boric acid/potassium chloride/sodium hydroxide) were provided by Merck (Germany).

### 2.2 Preparation of daunorubicin-containing antibubbles

The inner water phase (W_1_) for all four primary emulsions constituted 1 mL of pH 4 buffer containing 10% of maltodextrin (2 DE) and 2% daunorubicin mixed by manual shaking in a 2 mL Eppendorf tubes (Eppendorf, Germany). The oil phase consisted of 4 mL of cyclohexane containing hydrophobized fumed silica AEROSIL® R972 particles at concentrations of 1.5%, 2.5%, or 5% (Table 1). The dispersion in the oil phase was homogenized using sonication (BANDELIN SONOPLUS UV 2200, Germany) at 30% power for 30 s to get an even mixture while also preventing evaporation of cyclohexane at the same time. The inner water phase was added to the oil phase and homogenized using ULTRA-TURRAX® at 15,000 rpm for 1 min to prepare the W_1_/O primary emulsions. The outer water phase consisted of 16 mL of distilled water containing 5% maltodextrin (9 DE), 15% d-mannitol, 0.5% fumed silica AEROSIL® R816, and 0.5% AEROSIL® R972 for three formulations, while one formulation included only the relatively mildly hydrophobic particles, i.e., 1% AEROSIL® R816 instead (Table 1). The dispersions of the outer aqueous phases (W_2_) were homogenized with ULTRA-TURRAX® at 5000 rpm and sonication at 30% power for 40 s. The process was repeated thrice until all foam disappeared, all particles were dispersed in the aqueous phase, and a slightly opaque dispersion was obtained. The double emulsions (W_1_/O/W_2_) were prepared by adding the primary emulsions into the external water phases and homogenizing using ULTRA-TURRAX® at 7000 rpm for 40 s.

**Table 1:** Double emulsion and corresponding antibubble variants produced in this study.

Double emulsion and antibubble variants	R972 particle conc. in the oil phase	Particle type and conc. in the outer water phase
V1	1.5%	R816 (0.5%) + R972 (0.5%)
V2	2.5%	R816 (0.5%) + R972 (0.5%)
V3	5.0%	R816 (0.5%) + R972 (0.5%)
V4	2.5%	R816 (1.0%)

**Fig. 1:**
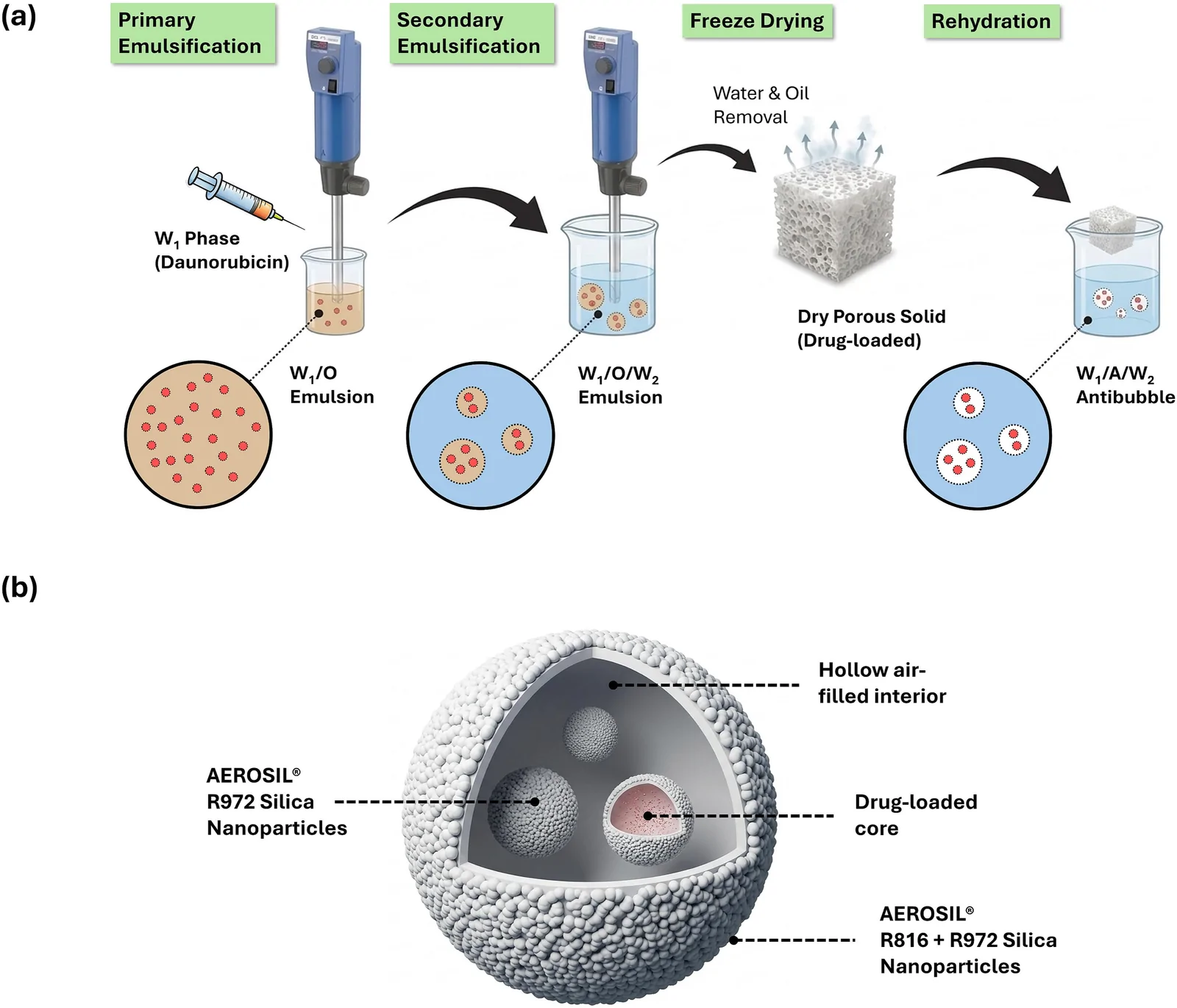
Schematic illustration of the formation and structure of particle-stabilized antibubbles. (a) The drug-containing inner aqueous phase (W_1_) is first emulsified in cyclohexane (oil phase) to form a W_1_/O emulsion, which is then dispersed in an external aqueous phase (W_2_) to obtain a W_1_/O/W_2_ double emulsion. Freeze-drying removes water and cyclohexane, yielding a dry porous matrix that forms antibubbles (W_1_/A/W_2_) upon rehydration. (b) Schematic structure of an antibubble with a hollow air-filled interior containing drug-loaded aqueous cores stabilized by silica nanoparticles.

The emulsion samples were placed instantly in a − 80 °C ultra-freezer (BINDER GmbH, Germany). An ultra-freezer was required to freeze samples quickly to minimize creaming and preserve the internal structure of double emulsions. The frozen product was then subjected to freeze drying (Lyovapor™ L-300, BUCHI, Switzerland) for 24–30 h at −80 °C with a vacuum of 0.01 mbar. This removes the volatile oil from the middle layer and water from both the water phases and converts the structure into a glassy state. The antibubbles were obtained by rehydrating the freeze-dried mixture with a suitable solution. The overall formation process and resulting antibubble structure are schematically illustrated in Fig. 1.

### 2.3 Characterization of antibubbles

#### 2.3.1 Entrapment efficiency

Entrapment efficiency (EE) of daunorubicin in the antibubbles was determined by quantifying the drug present in the external aqueous phase after rehydration and comparing it to the total drug content. The total daunorubicin concentration was measured after complete disruption of the antibubble structure induced by a surface-active agent (i.e., Tween 20). To prepare the sample, 0.25 g of freeze-dried powder was rehydrated in 10 mL distilled water that contained 10% maltodextrin solution to decrease the chance of structural breakdown due to osmotic pressure imbalances. The rehydrated sample was allowed to stand for a few minutes to enable the low-density antibubbles to rise, after which the lower aqueous phase was carefully sampled without disturbing the floating antibubble layer. An aliquot (0.5 mL) of the clear solution was withdrawn using a sterile syringe from below the antibubble foam and centrifuged at 2500 rpm for 5 min to allow any suspended particles to settle. Subsequently, 300 μL of the clear supernatant was carefully removed and mixed with 300 μL of pH 2 buffer (to ensure daunorubicin remains in protonated form). The mixture was vortexed for 10 s, and the absorbance was measured in a 96-well plate (Greiner Bio-One GmbH, Austria) at 480 nm using a spectrophotometer (Epoch microplate spectrophotometer, BioTek, USA).

To determine the maximum possible drug release, Tween 20 was added to the original antibubble suspension at a concentration of 1% of mixture, vortexed, and then allowed to stand for additional 5 to 10 min to completely destabilize the particle-stabilized interfaces. A sample from this suspension was centrifuged to obtain a clear supernatant, after which absorbance measurements were performed (as described in the above paragraph). The entrapment efficiency was calculated using the following equation:

(1) $$\mathrm{EE}\;\left(\%\right)=\frac{C_{\max}-C_{i}}{C_{\max}}\times 100$$

where, $C_{\max}$ is the daunorubicin concentration obtained after completely disrupting the antibubbles with Tween 20. $C_{i}$ is the initial daunorubicin concentration in the external aqueous phase after rehydration. The concentration of daunorubicin was quantified using a calibration curve in the range of 5–100 μg/mL, where absorbance exhibited a linear relationship with drug concentration in pH 2 buffer.

#### 2.3.2 Microscopic observation and image analysis

The microscopic images of double emulsions were recorded using an optical microscope equipped with a digital camera (Olympus, Japan). The antibubbles were also observed after rehydration in a 10% maltodextrin solution. The double emulsions and antibubble suspensions were placed on a large cavity glass slide and then visualized under the microscope at 10× and 20× magnifications.

The size of the antibubbles was recorded using images taken by the optical microscope. ImageJ software (Schneider et al., 2012) was used to calculate the antibubbles' size. The size of 500 antibubbles was measured for each measurement, and then the average size and standard deviation were calculated.

### 2.4 Factors controlling drug release from antibubbles

#### 2.4.1 Effect of pH

Freeze-dried V3 antibubble powder (0.27 g) was dispersed in pH 2, 5, and 7 buffers (each 10 mL) by gently manually shaking test tubes. Samples were collected at 0, 0.5, 1, and 2 h after dispersions were allowed to stand for 2 min and then analyzed in the same manner as described in Section 2.3.1. Since daunorubicin solubility and extinction coefficient in spectrophotometric analysis vary with pH, all samples were analyzed after adjusting pH to 2 to ensure consistent detection.

#### 2.4.2 Effect of bile salts

Freeze-dried V3 antibubble powder (0.27 g) was dispersed in 10 mL of pH 7 buffer containing either 0.5% (*w*/*v*) bile extract or sodium taurocholate (NaTC) at 5 or 20 mM. These concentrations were selected to reflect physiologically relevant intestinal bile salt levels, with 5 mM representing a lower physiological level and 20 mM representing an upper fed-state level (Santos et al., 2025). The dispersions were gently mixed and allowed to settle before analysis, while samples were taken at 0, 0.5, 1, and 2 h. Daunorubicin was quantified in the samples following the detection procedure described in Section 2.3.1, ensuring consistency across all measurements.

### 2.5 Effect of particles' concentration on drug release from antibubbles

Different formulations with various particle concentrations at the inner interface and particle types at the outer interface were evaluated to optimize the antibubble formulation for maximum protection against drug leakage in the gastric environment. Four antibubble formulations (as already presented in Table 1) were tested in pH 2 buffer (simulating gastric pH conditions). The samples were gently mixed and allowed to settle before analysis, and the drug release was recorded at 0, 0.5, 1, and 2 h. Daunorubicin was quantified following the detection procedure described in Section 2.3.1. Since the pH of all samples was already 2, no additional pH adjustment was required before spectrophotometric analysis.

### 2.6 Drug release from optimized antibubble formulation under simulated gastrointestinal conditions

The release of daunorubicin from the antibubbles was determined in simulated gastric fluid (SGF) and simulated intestinal fluid (SIF). SGF was an aqueous mixture of pepsin (2 mg/mL) and NaCl (2 mg/mL), whose pH was adjusted to 1.5 using 5 M HCl. SIF was an aqueous mixture of monobasic potassium phosphate (6.8 mg/mL) and pancreatin (5 mg/mL), whose pH was adjusted to 6.8 using 1 M NaOH. Additionally, SIF was supplemented with bile extract 0.5 (wt%) (Santos et al., 2025).

The daunorubicin-loaded antibubble powder (0.33 g) was added to a conical tube (50 mL) containing 10 mL of SGF (preheated to 37 °C). The samples were capped and placed in a shaking incubator (Binder, USA), rotating at 100 rpm, and maintained at 37 °C. Aliquots of samples were withdrawn at different time points (5 min, 1, and 2 h), and their absorbance was recorded at 480 nm to quantify the released daunorubicin (Section 2.3.1). After completion of gastric phase (i.e., 2 h), the SGF mixture containing the antibubble suspension was transferred to an equal volume of SIF. After mixing SGF with SIF, the pH of the medium was readjusted to ∼6.8 using 1 M NaOH. The release of daunorubicin was quantified by measuring the absorbance at 0, 2, and 4 h after transition to SIF, where 0 h corresponds to immediately after addition to SIF. All absorbance measurements were blank-corrected using the corresponding release medium without daunorubicin, including the bile extract-containing medium.

After completion of the release experiment, the mixture was treated with Tween 20 (at a concentration of 1% of the mixture) to attain the maximum drug release. The total drug release was then determined from the absorbance, as already described. Cumulative release (CR) of the drug was calculated using Eq. 2:

(2) $$\mathrm{CR}\;\left(\%\right)=\frac{C_{s}}{C_{\max}}\times 100$$

where, $C_{s}$ and $C_{\max}$ are the cumulative drug release and total drug concentration at a specific time.

### 2.7 Preservation of encapsulated daunorubicin by antibubbles

To evaluate the protective effect of drug-loaded antibubbles, the stability of encapsulated daunorubicin was assessed at two storage conditions, pH 9 at 4 °C and pH 9.5 at 23 °C, where daunorubicin is known to be unstable (Beijnen et al., 1986; Respaud et al., 2013). Pure daunorubicin solution (10 mL each with a concentration of 100 μg/mL) at both conditions was also kept alongside to monitor the degradation of pure drug. Daunorubicin-loaded antibubbles (0.31 g each) were added to water (whose pH was adjusted to 9 or 9.5 with 1 M NaOH). Samples from free daunorubicin solutions and drug-loaded antibubble solutions (from below antibubbles cake) were collected at 0 and 24 h and analyzed following the procedure outlined in Section 2.3.1. Samples examined at 4 °C were stored in the refrigerator throughout the experiment (except during sampling) to prevent additional degradation caused by light or temperature.

### 2.8 Data analysis

All experiments were conducted in triplicate. Statistical analysis and graph preparation were performed using GraphPad Prism 10.0.3 (GraphPad Software, Inc., USA). Where applicable, the effects were analyzed using analysis of variance (ANOVA), and means were compared using Tukey's test. All evaluations were conducted at a 5% significance level.

## 3 Results and discussion

### 3.1 Formation and characterization of emulsions and antibubbles

**Fig. 2:**
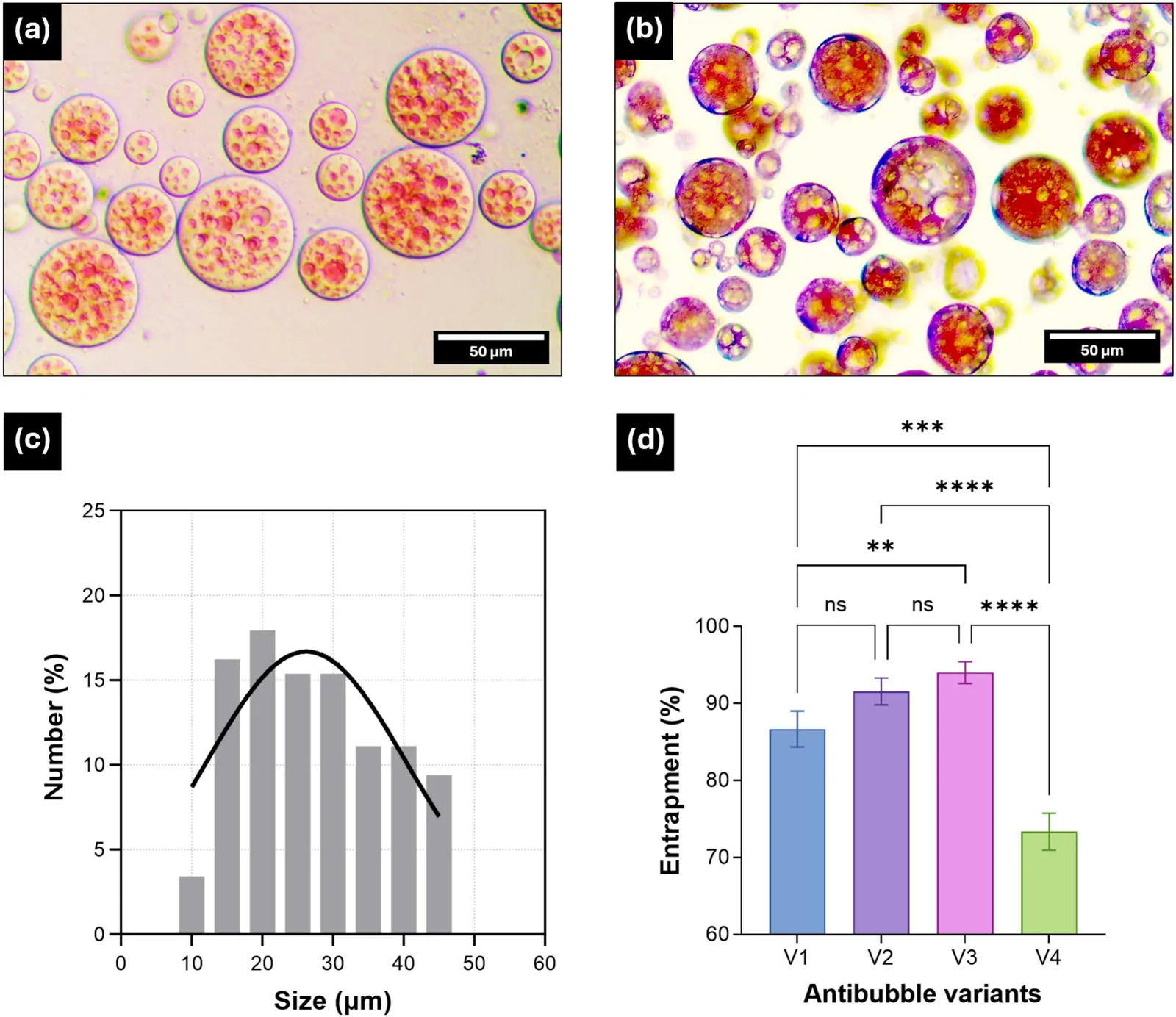
Daunorubicin-loaded formulations: (a) parent double emulsion (V3, Table 1), (b) rehydrated antibubbles at neutral pH (V3), (c) size distribution of antibubbles (V3), and (d) entrapment efficiency of different antibubble variants (V1, V2, V3, and V4, Table 1), where the error bars represent standard deviation (*n* = 3).

In this study, the term “parent double emulsion” refers to the W_1_/O/W_2_ emulsion before freeze-drying, whereas “antibubbles” refers to the W_1_/A/W_2_ structures obtained after freeze-drying and rehydration. The microscopic image of the parent double emulsion (Fig. 2a) of formulation V3 (Table 1) depicts most of the droplets as perfectly spherical, having bright, orange-colored inner cores conferring the presence of daunorubicin. After freeze-drying and rehydration, the corresponding antibubbles retained a multicore structure, indicating the presence of daunorubicin-containing aqueous cores within the antibubbles (Fig. 2b). The mean diameter of the final antibubbles after reconstitution was 25.7 μm (Fig. 2c). The entrapment efficiency (Fig. 2d) of all antibubble variants (V1, V2, V3) shows an increasing trend when the concentration of AEROSIL® R972 particles increases in the oil phase during double emulsion formation. V1 and V3 showed a statistically significant difference in entrapment efficiencies, i.e. 86% and 94%, respectively. A higher particle concentration at the oil-water interface results in a denser interfacial layer, which effectively inhibits droplet aggregation and coalescence, thereby maintaining smaller droplet sizes and enhancing the drug-encapsulating ability of the Pickering double emulsion (Frelichowska et al., 2009; Ribeiro et al., 2023; Yin et al., 2025). A high particle concentration could also be linked with forming an aerogel network inside the antibubbles, keeping the inner droplets of the antibubbles confined in space, as seen in particle-stabilized emulsions (Poortinga and Van Nostrum, 2025).

A notable decrease in entrapment efficiency was observed for V4 when relatively hydrophilic particles, AEROSIL® R816, were used alone at the outer interface, as depicted in Fig. 2d. Tukey's test revealed a significant difference in the entrapment efficiencies of V4 compared to V2 (91% vs. 73%), despite having the same particle concentration at the inner interface. This indicates that, during double emulsion formation, the interfacial adsorption of particles in V4 formulation is relatively slow, allowing escape of some inner droplets, likely due to the lower affinity of relatively hydrophilic particles (R816) for the oil-water interface than the outer water phase. In contrast, V2, with a combination of hydrophobic (R972) and relatively hydrophilic (R816) particles, ensures rapid interface establishment during the second emulsification step.

### 3.2 Factors responsible for drug release from daunorubicin-loaded antibubbles

#### 3.2.1 Effect of pH and bile extract

**Fig. 3:**
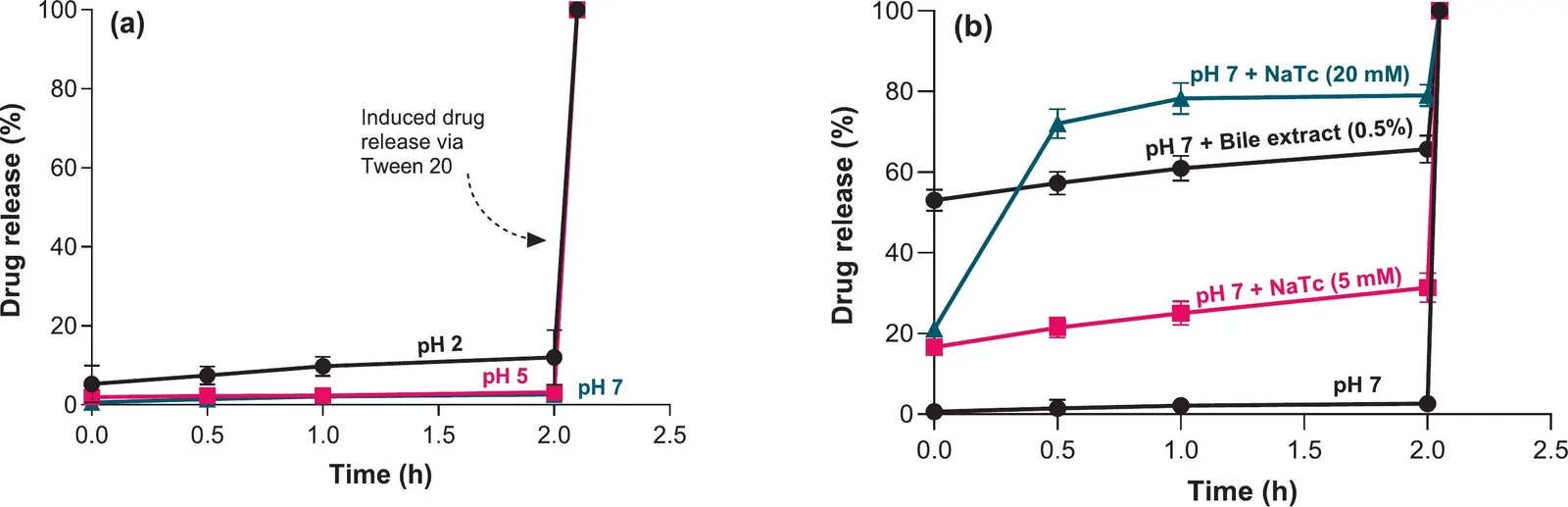
Daunorubicin release from antibubbles of type V3 (Table 1) under varying rehydration conditions: (a) Effect of pH (2, 5, and 7) on drug release, and (b) Effect of different concentrations of sodium taurocholate (NaTc) and bile extract on drug release. After two hours, Tween 20 (at 1% of the mixture) was added to break antibubbles for maximum drug release. The error bars represent standard deviation (*n* = 3).

The results presented in Fig. 3a demonstrate that the initial daunorubicin detected after rehydration of antibubbles remained low at all tested pH values (2, 5, and 7). This initial fraction corresponds to the non-encapsulated drug fraction indicated by the entrapment efficiency results in Section 3.1. The subsequent time-dependent release remained limited, confirming the stability of the silica nanoparticle-stabilized antibubbles under varying pH conditions. The slightly higher release at pH 2 is more likely due to increased daunorubicin solubility under acidic conditions, as has been reported for doxorubicin systems (Jafarzadeh-Holagh et al., 2018; Sultan et al., 2023), rather than pH-induced antibubble destabilization. These findings indicate that pH was not an effective trigger for drug release from the silica-stabilized antibubbles, whereas acidic pH can destabilize CaCO_3_-based antibubbles and trigger drug release (Zia et al., 2023).

Fig. 3a also shows a complete drug release upon the addition of Tween 20. The addition of Tween 20 displaces the silica particles at the interface, causing the collapse of the antibubble structure and subsequent release of its content. This forced release further supports that most of the drug was retained inside a protected antibubble structure and became available only after disruption of the particle-stabilized interfaces. For accurate quantification, the residual daunorubicin binding to silica after Tween 20 treatment was also considered. Although daunorubicin binding to silica was ∼35% before treating the mixture with Tween 20, this decreased to ∼5% after Tween 20 addition (as detailed in Supplementary Information, Section S.1). Since the total releasable drug was determined after Tween 20 treatment, only this residual ∼5% bound fraction was relevant for correction of the release percentages.

Fig. 3b demonstrates the critical role of bile salts (bile extract or NaTC) in destabilizing antibubbles and releasing daunorubicin. The drug release was minimal (less than 5%) when antibubbles were rehydrated in pH 7 alone. However, as bile salts were introduced, drug release increased significantly. NaTC impacted the release in a concentration-dependent manner, with 20 mM NaTC inducing substantially greater release than 5 mM. As mentioned in Section 2.4.2, these concentrations were selected to reflect physiologically relevant intestinal bile salt levels (Santos et al., 2025). Notably, bile extract (0.5%) produced the highest initial burst release, whereas 20 mM NaTC resulted in the highest overall drug release, followed by 0.5% bile extract; in contrast, 5 mM NaTC gave an appreciably lower release.

This destabilizing effect of bile extract was further confirmed by additional microscopy experiments performed to identify the minimum effective concentration required for antibubble disruption over a longer incubation time (4 h). Fig. S1 (Section S.2 of supplementary information) shows microscopic images of antibubbles exposed to increasing concentrations of bile extract. A gradual morphological transition from well-defined spherical structures to irregular, elongated particles was observed, with complete collapse occurring at around 1.5 mM after 4 h incubation at 37 °C. This concentration corresponds to approximately 0.1% (*w*/*v*) bovine bile and indicates that the complete bile extract is a more potent destabilizing agent than NaTC alone. A similar effect was also examined for other individual bile salts, and sodium deoxycholate, sodium glycocholate, and sodium cholate also required higher concentrations of around 20 mM or more to induce similar effects (data not shown).

These observations are consistent with the known role of bile salts, which are naturally present in the human intestine but absent in the stomach, and play a critical role as biological emulsifiers in the digestion of dietary fats (Shulpekova et al., 2022). They reduce interfacial tension and promote fat disruption, essential for efficient lipid absorption. In the case of silica-stabilized antibubbles, bile salts destabilize the particle-stabilized silica interfaces, thereby triggering the release of encapsulated drugs. Our findings highlight the physiological relevance of bile salts as a natural trigger for intestinal drug release while confirming that pH changes are not responsible for destabilizing the silica-stabilized antibubble.

#### 3.2.2 Mechanism of drug release

**Fig. 4:**
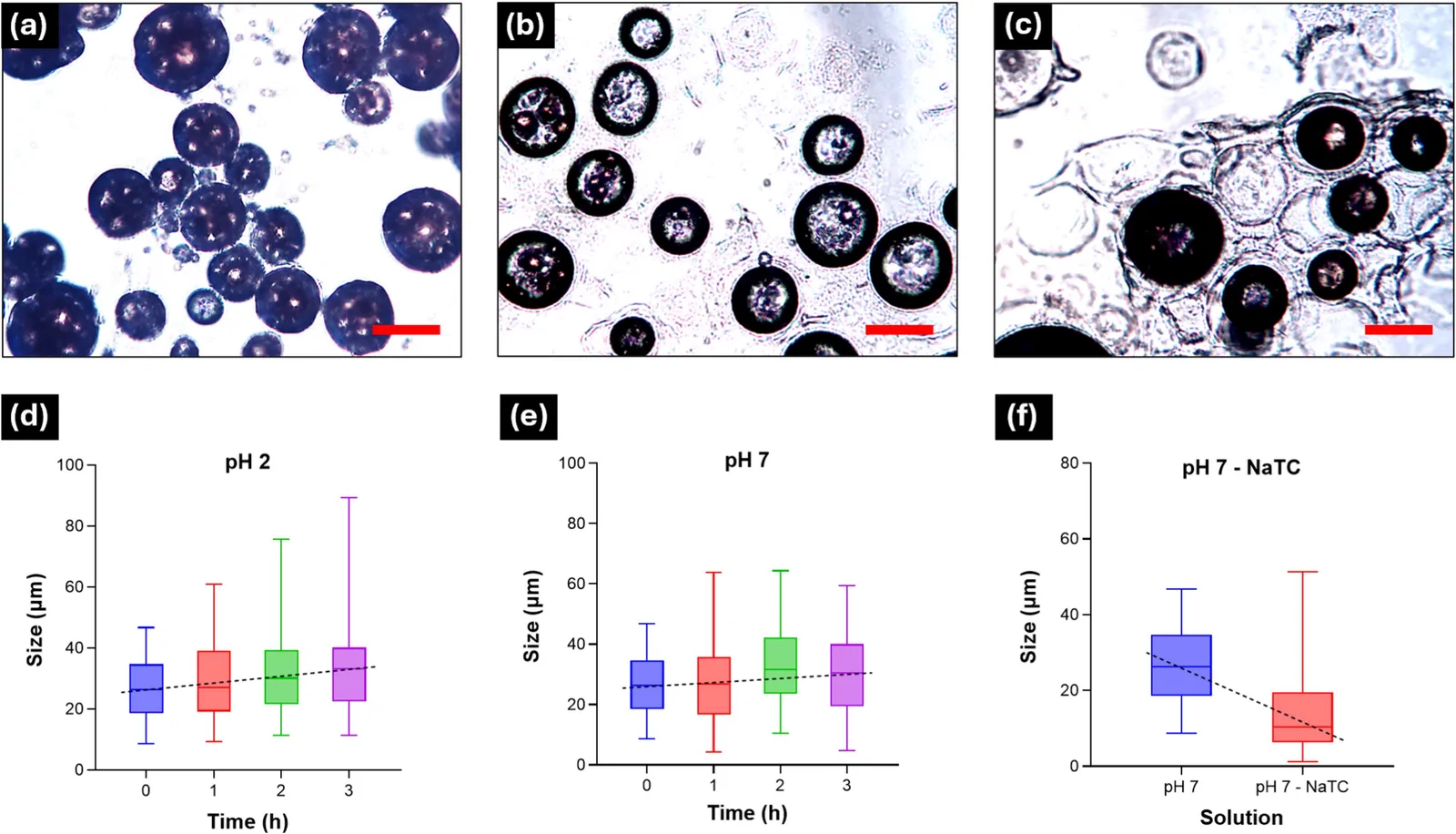
Microscopic images (top row) illustrating the structural changes in antibubbles (variant V3, Table 1): a) antibubbles after approximately 5 min of rehydration at pH 2, and b) & c) antibubbles upon sudden exposure of already rehydrated antibubbles to sodium taurocholate (20 mM) at pH 7, showing destabilization in antibubble structure. The scale bars represent 30 μm. The box plots (bottom row) show the size distribution from image analysis of antibubbles upon rehydration in different conditions: d) pH 2, e) pH 7, and f) after 10 min exposure to sodium taurocholate at pH 7.

To further visualize how bile salts affect antibubble structure, a higher NaTC concentration (20 mM) was used to more clearly observe the detailed structural changes associated with antibubble destabilization. Fig. 4b & c show the impact of NaTC on the structural integrity of antibubbles compared to when they were rehydrated in pH 2 (Fig. 4a) or pH 7 (Fig. 2b). The antibubbles remain intact upon rehydration at pH 2 and pH 7, exhibiting well-defined structures with inner cores. Sudden changes in antibubbles' appearance were observed upon exposure to 20 mM of NaTC. In Fig. 4b, the antibubbles exhibit fewer or no inner cores immediately after NaTC exposure, indicating rapid changes (disruption) of the internal structure. Fig. 4c specifically shows some residues and imprints of disappearing antibubbles. These microscopic observations were further validated through size measurements of antibubbles under varying pH conditions and in the presence of NaTC. Fig. 4d demonstrates that antibubbles in pH 2 buffer exhibit minimal changes in size over 3 h, with only a slight increase in mean size, showcasing the stability of silica-based antibubbles in acidic conditions. Similarly, antibubbles rehydrated in pH 7 buffer (Fig. 4e) showed a minimal increase in mean size and standard deviation over time. In contrast, Fig. 4f reveals that rehydration in a NaTC solution led to a marked and rapid decrease in antibubble size, reflecting the impact of NaTC on silica-stabilized interfaces and the resulting reduction of mean antibubble size.

These observations can be interpreted considering how surfactants interact with particle-stabilized interfaces. Surfactants like Tween 20 can disrupt particle-stabilized interfaces either by displacing the particles entirely to occupy the interface themselves or by adsorbing onto the particle surface, increasing their wettability and promoting their detachment from the interface (Vashisth et al., 2010). As NaTC is also a surface-active agent, it can similarly destabilize the antibubbles. Indeed, the structure collapse (drug release rate) depends on NaTC concentration, suggesting a rapid or partial (but gradual) destabilization of the antibubbles as a function of NaTC concentration. The antibubbles became relatively small and contained fewer inner cores immediately after treatment with NaTC. NaTC likely caused the initial detachment of some particles from the interface, resulting in localized defects (e.g., cracks) or a more porous interface. The phenomenon of crack formation in particle-stabilized interfaces was explained in detail by Vella et al. (2006). They explained that a local surface tension reduction occurs when a surfactant is introduced (with the help of a needle) on a densely packed monolayer of colloidal particles. That results in a tensile stress at the interface, creating a crack in the particulate layer. So, whatever the outcome of the interaction between antibubbles and NaTC (i.e., the formation of either cracks or disruption of particles from the interface), this would cause leakage of the entrapped air, leading to a reduction in antibubble size. Consequently, the external phase (containing NaTC) inrushes to the antibubble to replace the gap created by air leakage. Hence, the inner cores also begin to coalesce with the outer aqueous phase and subsequently disappear. Here, it is essential to note that the remains of the antibubbles are smooth and spherical in the presence of NaTC (Fig. 4b & c) as compared to when they were rehydrated in pH 2 or pH 7. In fact, the interface behaves initially like a solid due to a layer of adsorbed close-packed particles. After adding the bile salt, it behaves like a traditional air-liquid interface because the bile salt (NaTC) displaces (part of) the adsorbed particles. This could explain the increased sphericity of the antibubbles in the presence of NaTC.

#### 3.2.3 Impact of inner interface particle concentration and outer interface particle type on the release of daunorubicin at pH 2

**Fig. 5:**
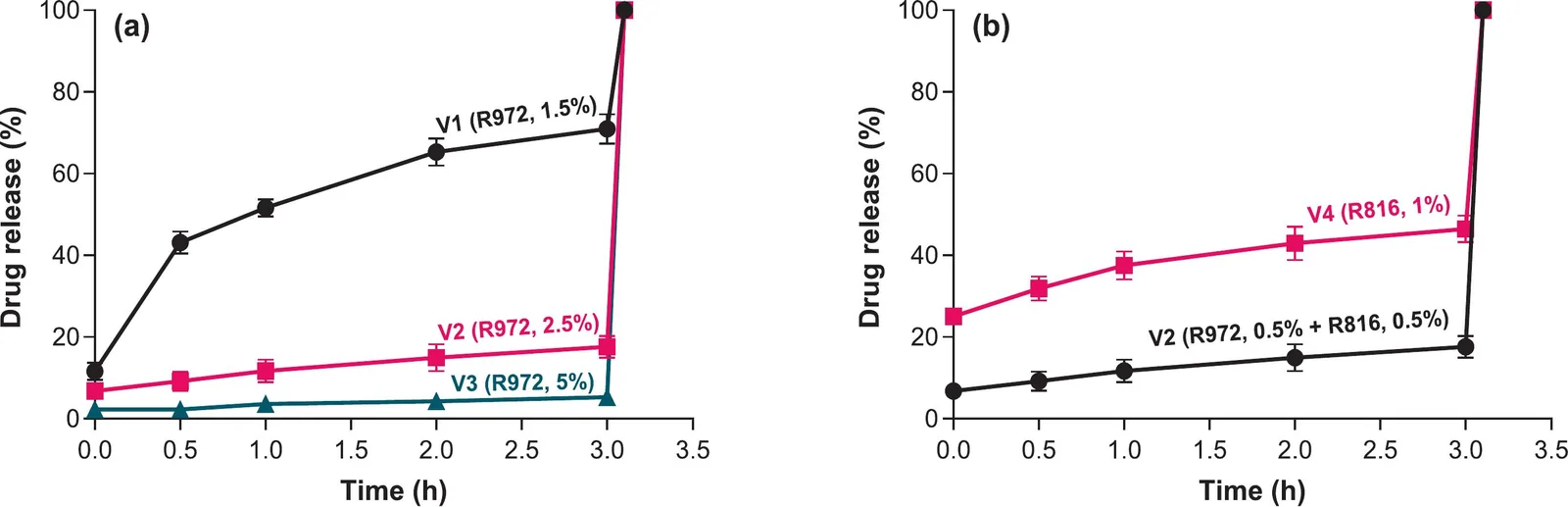
Daunorubicin release from antibubbles in pH 2 buffer as influenced by the type and concentration of AEROSIL® functionalized silica nanoparticles used during the fabrication of antibubbles: (a) Effect of varying concentrations of R972 particles in the middle oil phase (cyclohexane) of parent double emulsion and (b) Effect of different combinations of R816 and R972 particles in the outer aqueous phase of the parent double emulsions. After three hours, Tween 20 (at 1% of the mixture) was added to destroy the antibubbles entirely. The error bars represent standard deviation (*n* = 3).

The objective was to develop antibubbles that provide maximum protection for daunorubicin in the stomach while ensuring efficient release in the intestine. To achieve this, the formulation was optimized by varying the particle concentration at the inner interface and adjusting the particle composition at the outer interface (Fig. 5a & b, respectively). The release behavior was determined in a pH 2 buffer, mimicking the acidic gastric pH. Fig. 5a illustrates the release of daunorubicin from antibubbles prepared from emulsions with increasing concentrations of silica R972 particles in the oil phase. V1 variant (with the lowest silica content) exhibited significantly higher drug release compared to V2 and V3 variants, where minimal release was observed. The highest stability was achieved with the highest silica content (V3), where drug release remained below 5% even after 3 h in the pH 2 buffer. These results indicate that increased particle concentration at the inner interface enhances core stability and thereby prevents drug release. A higher particle concentration is associated with the formation of thicker and more cohesive interfacial layers, which provides better mechanical resistance against environmental stress, effectively trapping the core droplets within the gas shell. This behavior is consistent with previous observations in particle-stabilized foams and emulsions (Kargar et al., 2012; Lam et al., 2014b; Wu and Ma, 2016). Conversely, insufficient interfacial coverage, as seen with variant V1, leads to loosely packed particles resulting in a weaker interface.

While the inner interface of the antibubbles was stabilized with R972 silica particles, this is not possible for the outer interface because this led to poorly dispersible antibubbles. Therefore, the more hydrophilic R816 type of silica was introduced. Fig. 5b demonstrates the importance of outer interface composition in controlling drug release. Antibubbles with two different particle compositions at the outer interface were compared, i.e., with R816 alone (V4) or with a 1:1 mixture of R816 and R972 (V2), at a fixed particle concentration (2.5% R972) at the inner interface. At 0 h, the release with V4 starts at 25%, significantly higher than the 6% observed with V2. This difference corresponds to different entrapment efficiencies in Fig. 1d (Section 3.1). Then, after 3 h, the release for V4 approached 46%, whereas V2's release was still 18%. The release rates, calculated as the slopes of the linear regression, reveal a significant difference in cumulative drug release per hour i.e., from 3.5% for V2 (R^2^ = 0.98) to 6.8% for V4 (R^2^ = 0.92). A difference in release over some time of 3 h demonstrates that the synergistic effect of combining hydrophobic and hydrophilic particles at the outer interface (mainly due to a stronger interface) results in more robust antibubbles.

### 3.3 Simulated gastrointestinal drug release from antibubbles

**Fig. 6:**
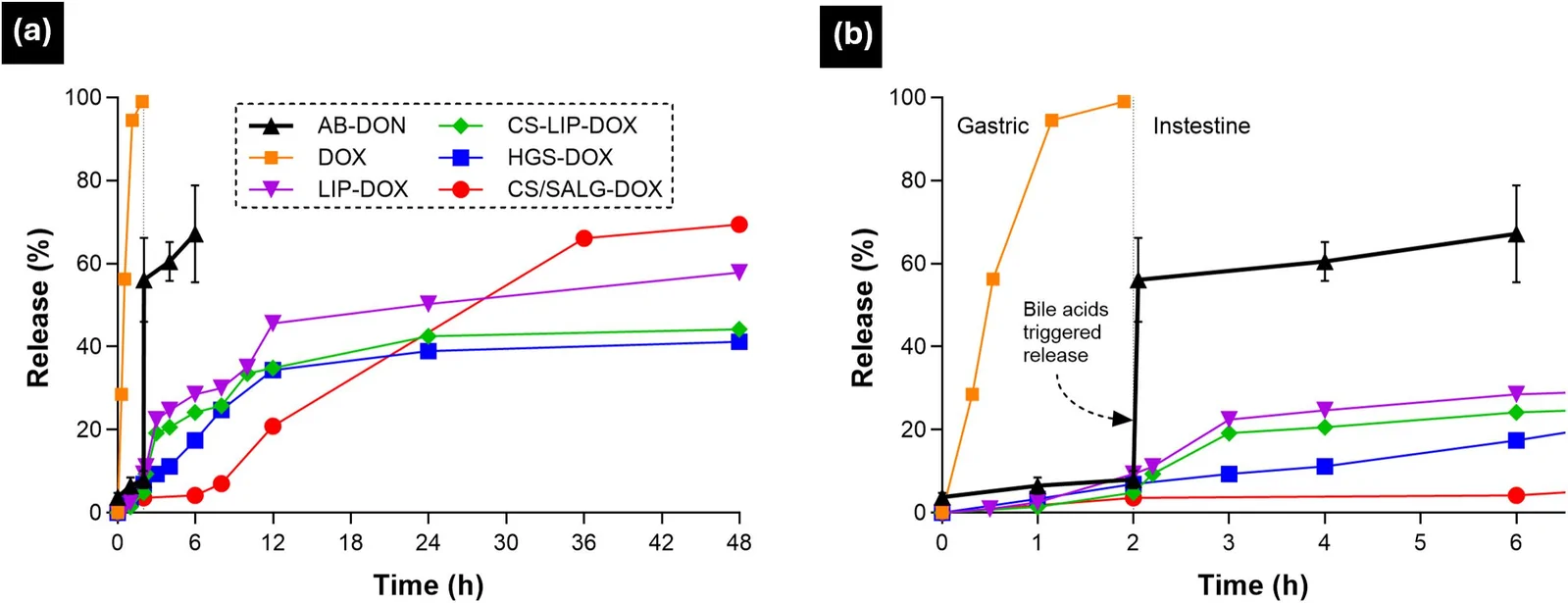
Comparison of drug release from daunorubicin-loaded antibubbles (black line) and selected oral doxorubicin formulations reported in the literature. (a) Release profiles up to 48 h, and (b) release profiles during the first 6 h, highlighting the gastric-to-intestinal transition. The error bars for the daunorubicin-loaded antibubbles represent standard deviation (*n* = 3), while literature data are shown as reported mean values. The details of the selected literature formulations are provided in Table 2.

To better position the release behavior of the present system, the release profile of daunorubicin from antibubbles (V3 variant) was compared with selected oral doxorubicin delivery formulations reported in the literature (Fig. 6). Direct studies reporting daunorubicin release under simulated gastrointestinal conditions could not be found; therefore, doxorubicin formulations were selected as the closest anthracycline-based systems for comparison. The selected systems included free doxorubicin solution, liposomal doxorubicin, taurine-chitosan-coated liposomal doxorubicin, hyaluronic acid-based doxorubicin nanoparticles, and chitosan/sodium alginate hydrogel beads, as summarized in Table 2. Most of these systems were designed either to improve oral absorption or to delay drug release toward the colon.

**Table 2:** Details of formulations used for comparison of release behavior in Fig. 6.

Code used in Fig. 6	Formulation name/type	Description/detail	Reference
AB-DON	Antibubbles	Silica nanoparticle-stabilized antibubbles loaded with daunorubicin	Present study
DOX	Free drug solution	Doxorubicin hydrochloride solution used as control	Qin et al. (2024)
LIP-DOX	Liposomes	Doxorubicin-loaded liposomes prepared from egg yolk lecithin and cholesterol	Qin et al. (2024)
CS-LIP-DOX	Coated liposomes	Doxorubicin-loaded liposomes coated with taurine-grafted chitosan	Qin et al. (2024)
HGS-DOX	Self-assembled nanoparticles	Doxorubicin-loaded hyaluronic acid-glycerol monostearate nanoparticles	Wu et al. (2024)
CS/SALG-DOX	Hydrogel beads	Doxorubicin-loaded double cross-linked chitosan/sodium alginate hydrogel beads; CS/SALG ratio 1:4	Wu et al. (2020)

During the gastric phase (0−2h), the antibubbles demonstrated excellent stability, with limited daunorubicin release (<15%). This indicates that the particle-stabilized interface remained largely intact under acidic conditions, in accordance with the stability behavior discussed in Section 3.2.3. However, a sharp increase in drug release occurred upon transitioning to SIF (supplemented with bile extract), with approximately 60% release observed immediately after exposure to the bile salts in the intestinal fluid. Afterwards, the release continued gradually, reaching nearly 80% after 6 h.

As discussed in Section 3.2.1, this burst release is mainly linked with the presence of bile salts in the intestinal phase. Bile salts, being amphiphilic molecules, interact directly with the silica particles at the air-water interface of the antibubbles (Zhang et al., 2021). The hydrophobic tails of the bile salts likely adsorb onto the hydrophobic domains of the silica particles while their hydrophilic heads remain oriented toward the surrounding aqueous medium. This interaction alters the particle wettability, reducing their affinity with the interface versus outer water. As a result, the silica particles detach from the antibubble structure, destabilizing and subsequently rupturing the antibubbles, thereby releasing the encapsulated drug. The effect of bile salts on an air-water interface was described comprehensively in another study (Torcello-Gómez et al., 2012).

Compared with the reported doxorubicin systems, the present antibubbles showed a distinct release pattern. The reported formulations generally exhibited a more gradual release, consistent with diffusion-controlled, sustained, or colon-targeted delivery. In contrast, the antibubbles retained most of the drug during the gastric phase and then released a major fraction upon transition to bile-containing intestinal fluid. Therefore, the main advantage of the present system is not only protection during gastric exposure but also the ability to achieve a bile salt-triggered burst release in the intestinal environment. Here, it should be noted that the intended function of antibubbles is not their uptake by the intestinal epithelium, but protection of daunorubicin during gastric exposure followed by its release in the intestinal lumen. Therefore, any subsequent absorption would involve the released daunorubicin, which should be further studied.

### 3.4 Preservation of encapsulated daunorubicin by antibubbles

As discussed in Section 3.2.2, antibubbles effectively maintain structural integrity and prevent the premature release of daunorubicin under gastric pH conditions. Nevertheless, an optimal encapsulation system should also ensure protection of the encapsulated drug from chemical degradation. To demonstrate this, we rehydrated antibubbles at pH 9 or above, a condition under which daunorubicin degradation has already been documented (Maniez-Devos et al., 1986; Piekarski et al., 2014). Although this extreme pH does not mimic the intestinal pH, it was selected to accelerate pH-dependent changes in daunorubicin structure, serving as a proof of concept for the ability of antibubbles to protect an encapsulated active from such changes. The degradation of daunorubicin was assessed spectrophotometrically by monitoring the decrease in absorbance at 480 nm, as described in Section 2.3.1.

**Fig. 7:**
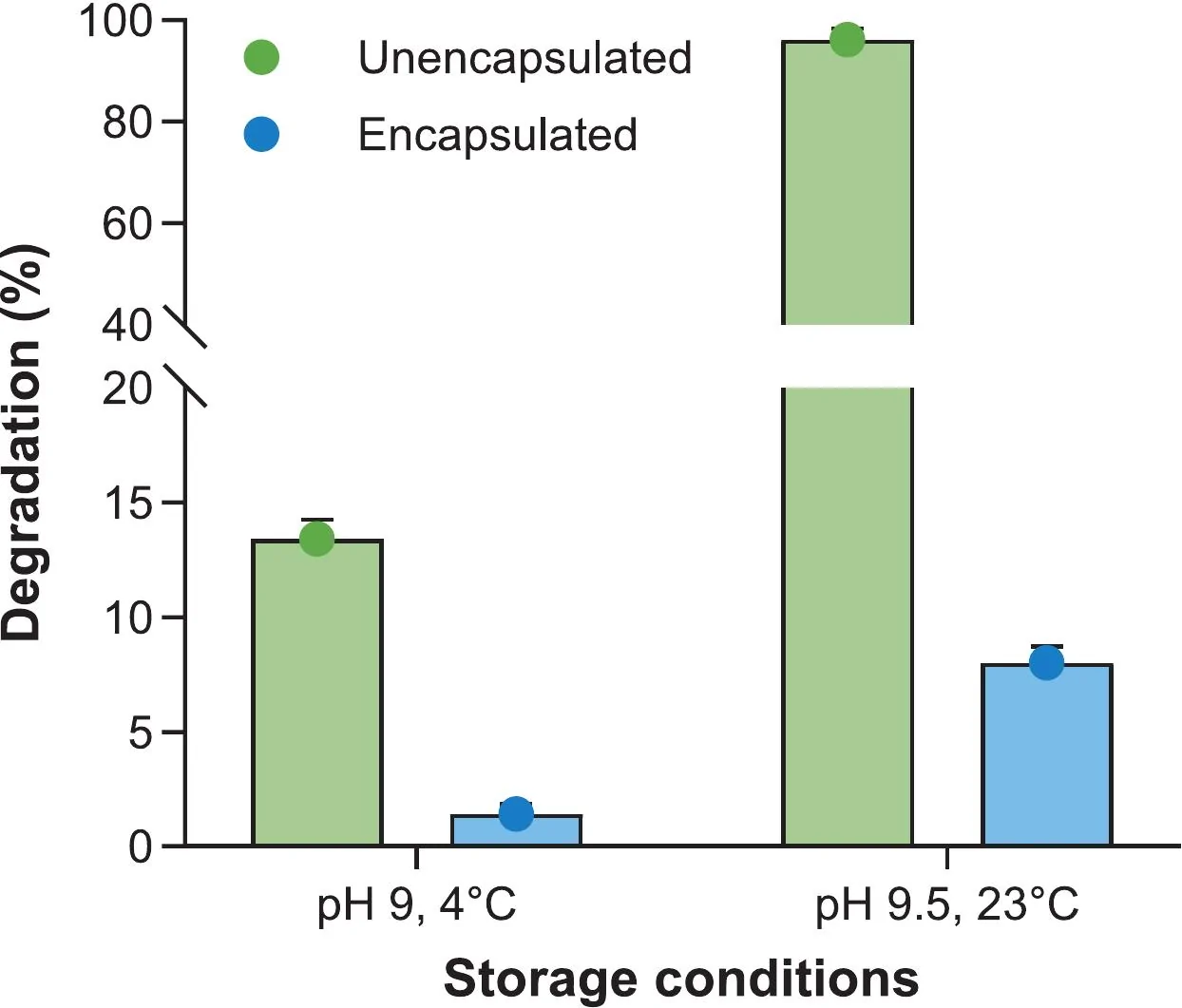
(a) Degradation (%) of unencapsulated (free drug in solution) and encapsulated daunorubicin (in antibubbles) at two storage conditions after 24 h. Data represent mean ± standard deviation (*n* = 3).

Fig. 7 depicts the degradation percentage of daunorubicin after 24 h under two different storage conditions: pH 9 at 4 °C in the dark and pH 9.5 at 23 °C at ambient light conditions, the latter representing a harsher storage condition. The comparison includes the degradation of pure (free) daunorubicin in aqueous solution and its encapsulated form within antibubbles. Under the more severe condition of pH 9.5 at 23 °C, the degradation of the free daunorubicin was around 90% in 24 h, corresponding to a pseudo-first order degradation rate constant (*k*) of 3.8 × 10^−5^ s^−1^. In contrast, the encapsulated drug showed significantly lower degradation, i.e., approximately 8% after 24 h. The latter value is close to the fraction of free drug present in the antibubble sample given the entrapment efficiency was around 94% (see Fig. 1d). A fair conclusion is then that the encapsulated fraction of daunorubicin is (almost) fully protected against degradation. At pH 9 at 4 °C, the degradation of the free daunorubicin reached around 12% (*k* = 1.7 × 10^−6^ s^−1^), while the encapsulated drug exhibited minimal degradation, approximately 1–2%, again most likely related to part of the free (unencapsulated) drug fraction in the antibubble sample.

For comparison, Piekarski et al. (2014) reported degradation rate constants of 7 × 10^−6^ s^−1^ and 2 × 10^−5^ s^−1^ at 40 °C for pH 9 and 9.5, respectively. Applying the general rule that degradation rates approximately halve with every 10 °C decrease in temperature, we extrapolated their values to estimate rate constants of 5.8 × 10^−7^ s^−1^ at pH 9 and 4 °C, and 6.16 × 10^−6^ s^−1^ at pH 9.5 and 23 °C. The experimentally observed rate constants in our study (1.7 × 10^−6^ s^−1^ and 3.8 × 10^−5^ s^−1^, respectively) are higher than these estimates but remain less than an order of magnitude different. This indicates that the values are reasonably comparable, with the observed differences likely stemming from variations in experimental conditions, such as light exposure at pH 9.5 and 23 °C, which may accelerate degradation. Overall, the comparison supports the consistency of our experimental data with established degradation behavior.

These findings demonstrate that antibubbles effectively protected daunorubicin from hydroxyl ion-induced degradation under both conditions, almost all of the drug remaining intact within the antibubble structure even under the severe conditions, where most of the free drug had degraded in solution within 24 h.

## 4 Conclusion

This study demonstrated the potential of silica nanoparticle-stabilized antibubbles as an innovative oral delivery system for chemotherapeutics (daunorubicin). The formulated antibubbles effectively encapsulated the drug, protecting against premature release in the gastric environment. The optimized formulation exhibited high entrapment efficiency (94%) and remained stable under varying pH conditions, ensuring minimal drug leakage. A key finding was the bile salt-triggered release mechanism, where bile salts acted as a natural stimulus, displacing interfacial silica particles and facilitating a burst release of daunorubicin in the intestinal environment. This property enables site-specific drug delivery while potentially reducing premature exposure of free daunorubicin in the upper GI tract. Furthermore, stability studies at pH 9 or above confirmed that almost all encapsulated drug remained intact after 24 h, compared to a 90% degradation rate observed for the free drug. These findings highlight the potential of antibubbles as a promising carrier for oral chemotherapeutic delivery, addressing key challenges in drug stability and targeted release. To further advance the application of such antibubbles for oral chemotherapeutic delivery, future studies should focus on dedicated biological evaluation, including mucosal cytotoxicity, oral tolerability, cellular uptake, and in-vivo validation. These studies will provide crucial insights into whether the reduced gastric release and bile salt-triggered intestinal release observed here can translate into lower mucosal toxicity and improved oral bioavailability compared with free daunorubicin.

## CRediT authorship contribution statement

**Rabia Zia:** Writing – original draft, Methodology, Investigation, Formal analysis. **Annemarije van der Vorst:** Investigation. **Albert T. Poortinga:** Writing – review & editing, Methodology, Conceptualization. **Akmal Nazir:** Writing – review & editing, Resources. **Cornelus F. van Nostrum:** Writing – review & editing, Supervision, Conceptualization.

## Declaration of competing interest

The authors declare that A.T.P. owns a patent on a similar encapsulation system, which may be considered a potential competing financial interest; there are no other competing interests.

## Data Availability

Data will be made available on request.

## Appendix A Supplementary data

Schematic illustration of the formation and structure of particle-stabilized antibubbles. (a) The drug-containing inner aqueous phase (W_1_) is first emulsified in cyclohexane (oil phase) to form a W_1_/O emulsion, which is then dispersed in an external aqueous phase (W_2_) to obtain a W_1_/O/W_2_ double emulsion. Freeze-drying removes water and cyclohexane, yielding a dry porous matrix that forms antibubbles (W_1_/A/W_2_) upon rehydration. (b) Schematic structure of an antibubble with a hollow air-filled interior containing drug-loaded aqueous cores stabilized by silica nanoparticles. IMAGE 1
