# Supplementary material for: Bile acid triggered release of daunorubicin from silica nanoparticle-stabilized antibubbles
Source: Int J Pharm X. 2026 Aug 9;12:100632. doi: 10.1016/j.ijpx.2026.100632 (PMC13499053; doi:10.1016/j.ijpx.2026.100632)
Supplement: Supplementary file 1 [file mmc1.pdf]

Supplementary Information

Bile acid triggered release of daunorubicin from silica nanoparticle-stabilized antibubbles

S.1. Assessment of daunorubicin binding to silica particles

During spectrophotometric quantification, adsorption of daunorubicin onto dispersed silica nanoparticles could potentially reduce the measured free drug concentration. Therefore, control experiments were conducted to quantify drug-silica adsorption under simplified conditions. Importantly, these experiments were designed solely to evaluate potential analytical interference and do not necessarily represent the structural mechanism responsible for drug retention within intact antibubbles.

Scenario 1: Drug-silica interaction in the absence of surfactant

This experiment evaluated drug adsorption onto dispersed silica particles under surfactant-free conditions. Two daunorubicin solutions (10 mL each, 50 µg/mL) were prepared. To one solution, AEROSIL® R816 was added at 1% (w/v) and dispersed by sonication (BANDELIN SONOPLUS UV 2200, Germany) at 30% power for 30 s. The mixture was allowed to stand for 10 min to permit drug adsorption onto silica particles. The second solution (without silica) served as control. Both samples were filtered through a 0.2 µm syringe filter to remove silica particles. The absorbance of the filtrates was measured at 480 nm using an Epoch microplate spectrophotometer (BioTek, USA). Drug concentrations were calculated using the calibration curve described in the main manuscript.

Scenario 2: Drug-silica interaction in the presence of Tween 20

This experiment assessed whether surfactant treatment was able to reduce possible drug-silica adsorption, which is relevant for the conditions used to determine the maximum release in Section 2.3.1. Two solutions (10 mL each) containing 50 µg/mL daunorubicin and 1% (w/v) Tween 20 were prepared. AEROSIL® R816 (1% w/v) was added to one solution and sonicated under the same conditions as described above. The second solution served as control. After equilibration, samples were filtered (0.2 µm) and analyzed spectrophotometrically at 480 nm as described above.

Results and interpretation

In the absence of Tween 20, approximately 35% of daunorubicin adsorbed onto dispersed silica particles. This adsorption reflects drug-surface interactions occurring in a freely dispersed silica system. However, this observation should not be interpreted as the primary mechanism governing drug retention in intact antibubbles. In the actual antibubble system, drug stabilization arises predominantly from the preserved

interfacial architecture of the silica-stabilized structure, which restricts drug release in the absence of surface-active agents.

Upon addition of Tween 20, drug adsorption decreased to approximately 5%, indicating that surfactant-induced changes in wettability weaken drug-silica interactions. More importantly, surfactant exposure disrupts the antibubble interface, leading to structural breakdown and subsequent drug release.

For accuracy, EE and drug release values reported in the main manuscript were corrected by accounting for the residual ~5% drug fraction that remained associated with silica even after Tween 20 treatment.

S.2. Effect of bile extract concentration on antibubbles integrity

Antibubble powder (V2, 1.5 wt%) was rehydrated in an aqueous solution containing 10% maltodextrin. Bile bovine extract (Sigma-Aldrich, United States) was diluted to obtain the bile salt concentrations ranging from 0.01 mM to 121.72 mM. These conjugated bile acid concentrations were calculated using an estimated average molecular weight of 452.6 Da, based on the characterization of the bile bovine extract by Hu et al. (1). The antibubbles were homogeneously dispersed in the maltodextrin by gently shaking, and samples were taken. Each dilution of the bile extract was added to one of the antibubble samples in an equal volume. All tubes were placed on a roller mixer for 4 hours, either at room temperature or at 37°C. The condition of the antibubbles was assessed using light microscopy.

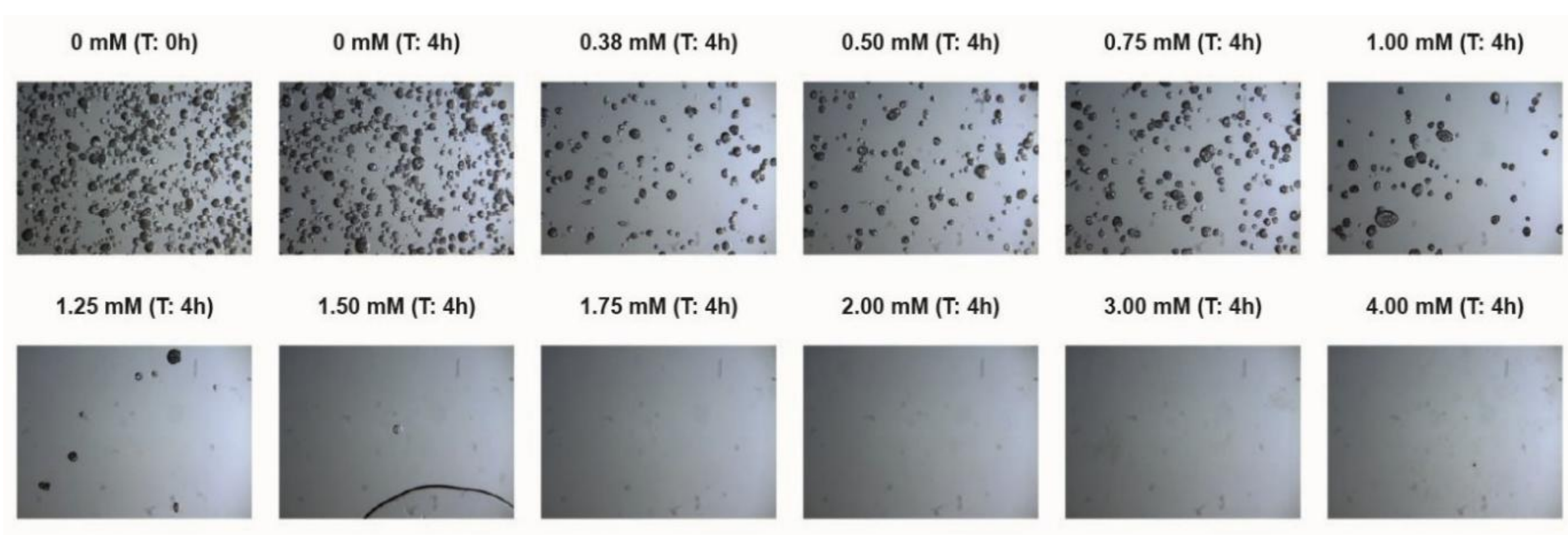

Fig. S1. Microscopic images of antibubbles before the addition of bile (T: 0h) and after 4 hours (T: 4h) at 37°C with increasing concentrations of bile extract. Bile extract concentrations of ≥1.5 mM triggered complete antibubble collapse.

References:

(1) Hu P-L, Yuan Y-H, Yue T-L, Guo C-F. Bile acid patterns in commercially available oxgall powders used for the evaluation of the bile tolerance ability of potential probiotics. PLOS ONE. 2018;13(3).
